# Predicting delayed antenatal care initiation among pregnant women in East Africa: using machine learning algorithms

**DOI:** 10.3389/fgwh.2025.1488391

**Published:** 2025-10-03

**Authors:** Nebebe Demis Baykemagn, Eliyas Addisu Taye, Mequannent Sharew Melaku, Tirualem Zeleke Yehuala, Makda Fekadie Tewelgne, Fetlework Gubena Arage, Adem Tsegaw Zegeye

**Affiliations:** 1 Department of Health Informatics, Institute of Public Health, College of Medicine and Health Sciences, University of Gondar, Gondar, Ethiopia; 2 Department of Epidemiology and Biostatistics, Institute of Public Health, College of Medicine and Health Sciences, University of Gondar, Gondar, Ethiopia

**Keywords:** ANC, machine learning, artificial intelligence, east Africa, pregnancy

## Abstract

**Background:**

Sub-Saharan Africa has the highest rate of maternal death due to pregnancy-related complications. The delayed onset of Antenatal care (ANC) is a major underlying factor for maternal mortality. The overall well-being and health of pregnant women and their fetuses greatly depend on the timely initiation of ANC care. This study aims to identify the top predictors of delayed antenatal care initiation in East Africa, including Ethiopia, to provide input for policymakers.

**Methodology:**

We employed secondary data from the Demographic Health Surveys conducted across ten East African countries between 2016 and 2023. After preprocessing the data, which included cleaning and feature selection through Recursive Feature Elimination, we addressed class imbalance using Synthetic Minority Over-sampling Technique (SMOTE). We then applied seven supervised learning algorithms to develop a robust machine learning model. The LGBM Classifier emerged as the top performer for predicting delayed antenatal care initiation, achieving accuracy of 75%, an AUC score of 81%, an F1 score of 78%, a recall of 79%, and a precision of 77%. Additionally, we employed association rule mining to further analyze.

**Result:**

Home delivery, low household income, a large number of household members, unemployment, being a younger woman, not using family planning methods, low educational level, and rural residence are predictors of delayed antenatal care initiation.

**Conclusion:**

The prevalence of late antenatal care (ANC) initiation is high (65%). Promote family planning utilization, targeted health literacy campaigns, youth-friendly programs, expand mobile clinics, and promote skilled birth attendance to increase timely ANC initiation are recommended.

## Introduction

One major concern for global health is the elevated incidences of maternal and infant mortality (1). Globally about 260,000 deaths in 2023, 92% of them preventable and mostly occurring in low- and middle-income countries for 180,000 and the highest lifetime risk at 1 in 49 woman (2, 3). Despite international efforts to lower maternal mortality, globally, pregnancy-related problems still kill thousands of women, with sub-Saharan Africa responsible for almost 70% of these deaths (4).

A WHO report showed that maternal death rates are highly discrepant between developing and developed nations, with rates of 430 and 13 per 100,000 live births, respectively (5).

The delayed onset of antenatal care (ANC) is a major underlying factor for maternal mortality (6). The overall well-being and health of pregnant women and their fetuses are greatly dependent on timely ANC care initiation (7). Preterm delivery and low birth weight are common results of delaying ANC check-ups (8). Globally, 75% of neonatal deaths are caused by preterm deliveries (9). Another consequence of missing ANC visits is anemia, often caused by low intake of iron and folic acid (10). With the right iron and folate supplements, pregnancy-related anemia can be effectively controlled and avoided (11).

The fundamental building block for delivering outstanding pregnant woman services is the timely implementation of promotion, preventive, screening, and diagnostic initiatives (12, 13). Those are strategies to realize the World Health Organization's (WHO) vision of quality health service for all pregnant mothers everywhere (12). Pregnancy-related services, such as ANC, improve and offer integrated service delivery with components related to prevention to mother to child transmission (PMTC) and HIV prevention (14). Due to the quality of ANC services, HIV-positive women can deliver HIV-negative infants (15).

Antenatal care is “the care of the woman during pregnancy” (16). Antenatal care has integrated pillars which include risk identification, prevention, and management, and health promotion related to pregnancy (12). Early prenatal care visits are vital for improving the physical, mental, and social well-being of women, and their children (17). Essential antenatal care services like birth planning, emergency preparedness, and childbirth readiness are vital to ensuring positive outcomes for women and their babies (18). A balanced diet is essential for women's health during pregnancy to promote healthy fetal growth and development, avoid stunting and wasting, and ensure great pregnancy outcomes (19).

Also, one of the WHO's strategies for reducing maternal and perinatal deaths is to enhance early and ongoing antenatal care, which is recommended to include a minimum of eight visits (20). The first ANC care visit is a crucial benchmark for subsequent antenatal care. The first antenatal care visit, also known as the “early ANC visit”, should take place within the first 12 weeks of pregnancy (WHO). Evidence revealed that timely initiation with eight ANC contact visits reduced maternal mortality by 20% (20).

Early antenatal care provision helps prevent negative pregnancy outcomes like low birth weight, premature delivery, jaundice, and early detection and treatment of syphilis, anemia, and malaria (21). On-time ANC initiation enables women to recognize potential risk factors and enhance their nutritional status (22). Early initiation of ANC is the cornerstone of early identification and treatment.

However, the incidence of early prenatal care visits differs by 57.9% between rich and poor countries (23). According to a study conducted in 19 African nations, 37.15% of pregnant women had their first antenatal care (ANC) contact during the 14th gestational week (8). Furthermore, according to the 2016 EDHS, only 20% of expectant mothers received their first antenatal care (ANC) visit before reaching 16 weeks of gestational age (24). A systematic review study also showed that 64% of Ethiopian women delay seeking antenatal care during pregnancy (25).

The evidence indicated that there were notable regional differences in the early initiation of first antenatal care. Tanzania (76%) (26), New Zealand (17%) (27), Gambia (56%) (28), Afghanistan (66.9%) (29), Malawi (75.6%) (30), (44.8%), Mozambique (60%) (31), and parts of Ethiopia (47%, 58%, 59%, 63%, 66%, 70%, 71.2%, and 78.4%) (32–39).

Based on the evidence, factors such as negative attitudes, low literacy levels, unplanned pregnancies, and lack of awareness regarding the importance of early ANC visits, along with maternal educational status, age, marital status, economic status, place of residence, parity, exposure to mass media, and distance from health facilities, are common significant contributors to delayed ANC initiation (26, 30, 40–42).

To our knowledge, there is no published study using machine learning (ML) to investigate the late initiation of AN (algorithms, previous studies conducted within small study areas and small sample sizes are non-representative of generalizability. ML algorithms are used to assess large amounts of data to confirm the predictors' role in a given problem which is good for generalizability and used as input for health policymakers for decision-making (43).

## Materials and methods

### Study setting

East African countries' DHS dataset was used in this study. East Africa is one of the largest regions on the African continent, including Kenya, Tanzania, Uganda, Rwanda, Burundi, and parts of Ethiopia, South Sudan, and Somalia (44).

### Data source

The data on delayed antenatal visits was extracted from the Demographic Health Survey (DHS) 2016 to 2023 of ten East African countries, namely Burundi, Ethiopia, Kenya, Zimbabwe, Madagascar, Malawi, Mozambique, Rwanda, Tanzania, and Uganda, which were selected for this study based on the availability of recent DHS data and their high burden of Maternal mortality, as well as delays in the initiation of antenatal.

### Population and eligibility criteria

The source population for this study was all pregnant women (aged 15–49) in ten East African nations who received ANC visits. The study population consisted of women who began ANC after 14 weeks of gestation.

### Sample size determination and sampling technique

Using the most recent DHS data, we generated a weighted sample of 77,865 pregnant women who have ANC visits from the ten East African countries. The study used the standard two-stage stratified cluster sampling design employed by the DHS. In the first stage, enumeration areas (EAs) were selected randomly within each stratum using probability proportional to size. In the second stage, households within each selected EA were chosen through systematic random sampling. Eligible women aged 15–49 in the selected households were then interviewed using the DHS individual women's questionnaire

### Study variables and measurements

Delay initiation of antenatal care was the outcome variable, categorized as: 1 = Yes (first antenatal care initiation within the 16th week of gestational age or four months) and 0 = No (initiation after this time).

Predictor Variables adapted from different (33, 34, 36, 38, 45–48) which include Socio-demographic factors: Age of women, type of place of residence, highest educational level, number of household member, sex of household head, currently marital status, sons at home, Getting medical help for self, and distance from the health facility.

Socio-economic factors: mobile ownership, literacy, Respondent occupation, internet usage, Wealth index.

Maternal health service utilization-related factors: Current use by method type, the total number of children ever born, place of delivery, and delivery by CS.

### Operational definitions

**Delayed antenatal** care refers to starting the first ANC appointment after 4 months of gestational age (38).

**Machine learning** is an application of artificial intelligence that involves algorithms and data that automatically analyze and predict without human intervention (49).

### Data management and analysis

STATA version 17 and Microsoft Excel 2019 were used to clean and weighted the data. Python 3.9 was used with incorporating key packages such as Pandas, scikit-learn, matplotlib, scikit-plot, and others. These packages facilitated data preprocessing, splitting, feature selection, model training, and final outcome prediction (50).

We employed data analysis procedures that included pre-processing techniques such as data cleaning to eliminate any duplicates, missing values, or outliers from the dataset. We also used the seven steps of the machine learning framework (51).

### Data collection

For this study, data was gathered from ten East African countries to train the model and enhance its predictive accuracy. Specifically, data on delayed antenatal visits was extracted from the Demographic Health Surveys (DHS) from 2016 to 2023. The countries included in the study were Burundi, Ethiopia, Kenya, Zimbabwe, Madagascar, Malawi, Mozambique, Rwanda, Tanzania, and Uganda. These countries were chosen due to the availability of recent DHS data and their significant levels of maternal mortality and delays in initiating antenatal care.

### Data preprocessing

According to previous evidence, data quality significantly impacts machine learning (ML) model performance and can lead to incorrect interventions or decisions. To mitigate these risks, data cleaning is a critical issue in the machine learning process, as it helps ensure the reliability and accuracy of the model's outcomes (52).

**Figure 1:**
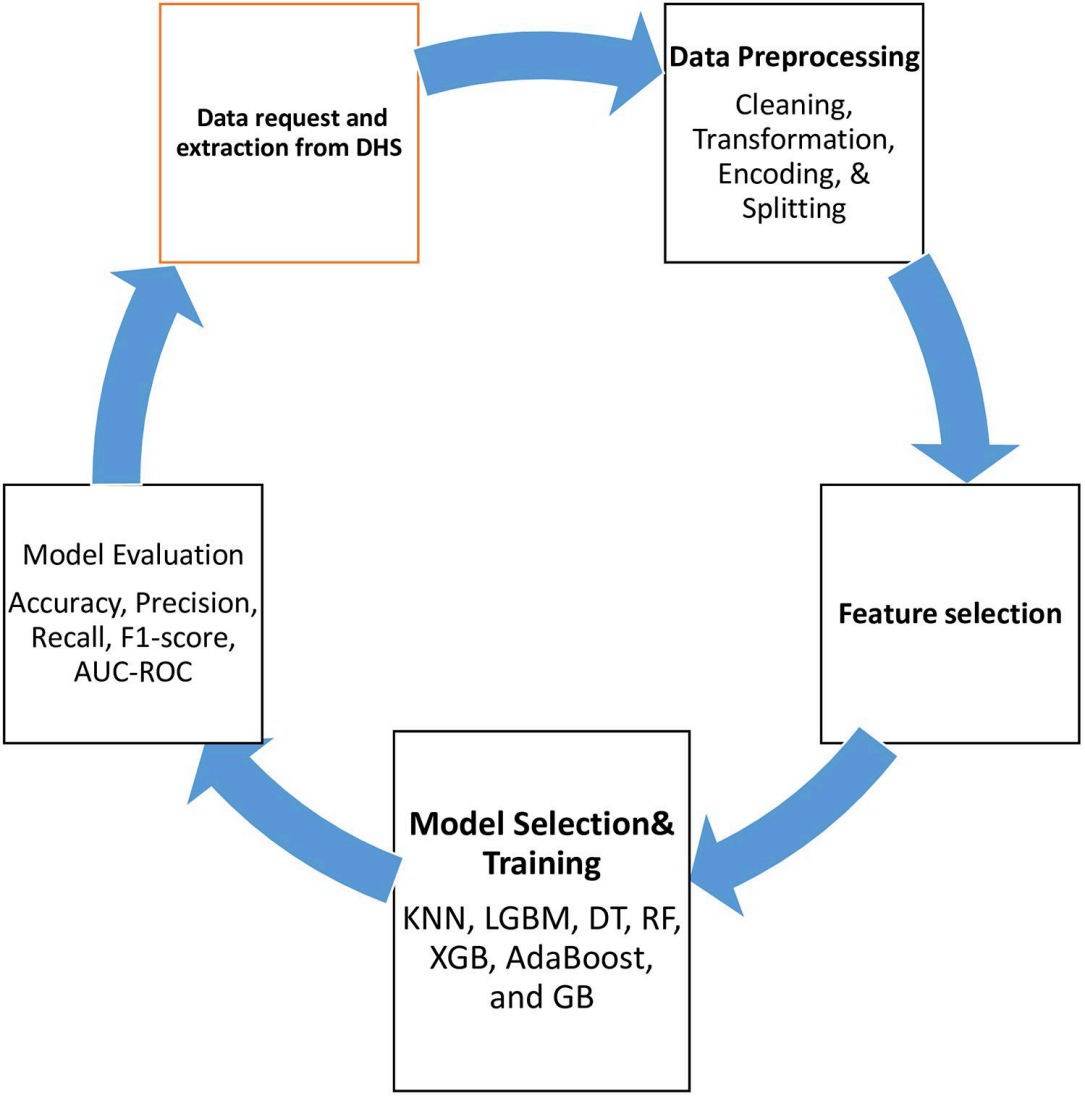
Overall workflow for prediction of ANC initiation.

In this study, we focus on comprehensive data-cleaning techniques essential for machine learning. We address missing values through various imputation methods, such as mean, median, and mode, and remove rows or columns with excessive missing data. For instance, missing age values are filled with the median age of the dataset. The interquartile range (IQR) approach and boxplot analysis were used to identify and handle outliers. Furthermore, we address outliers, normalize and standardize features, and use One-Hot Encoding, Min-Max Normalization, and Ordinal Encoding for categorical, numerical, and ordinal data, respectively. These preprocessing steps ensure that the data is appropriately formatted and ready for machine learning algorithms (53). As seen in Figure 1.

### Balancing dataset

**Figure 2:**
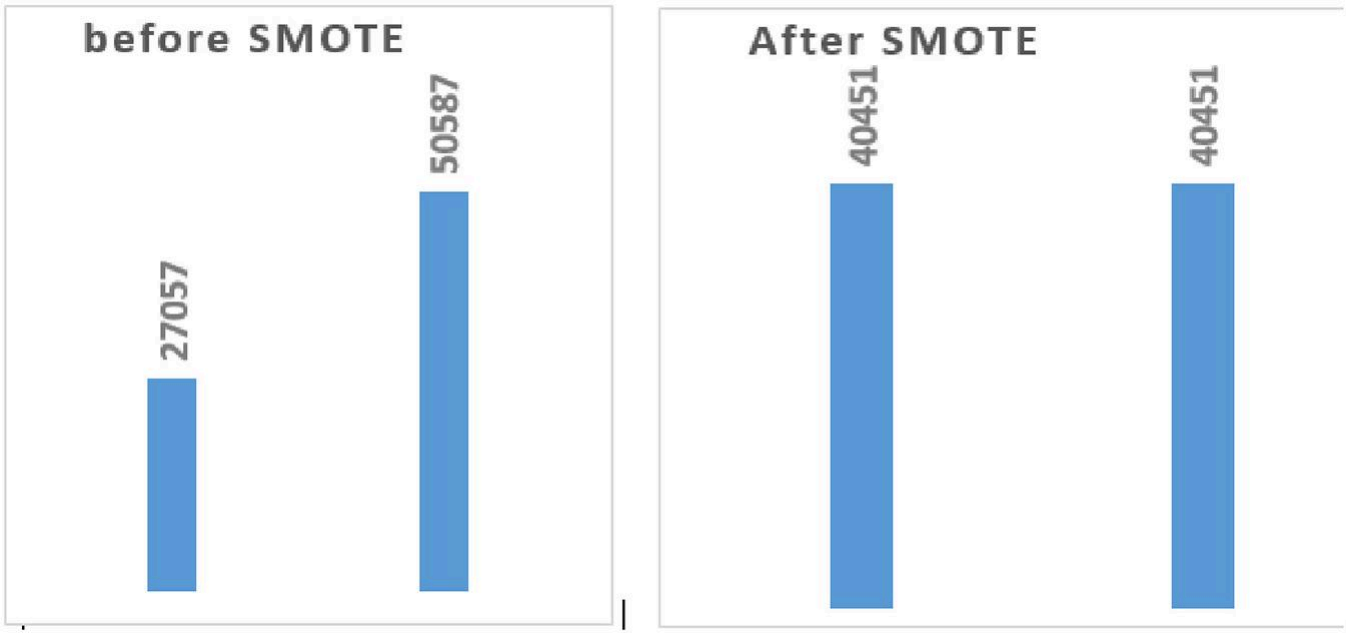
Data balancing using SMOTE.

Another essential stage in using machine learning models to make accurate predictions is balancing the data prior to model training (54). To prevent machine learning models from producing biased or unreliable results, especially those that perform poorly in the minority class (late initiation of antenatal care) For instance, the study data indicated “YES” (27,507) and “NO” (50,587). To balance this imbalance, we used the Synthetic Minority Over-sampling Technique (SMOTE) to increase both classes to 40,451 instances each. As seen in (Figure 2).

### Model selection

For this study, the dataset was divided into two subsets: 80% (6,473 samples) for training and 20% (1,618 samples) for validation and testing, using machine learning techniques.

Furthermore, since the data is intended for classification and is supported by previous research, we considered the following models: AdaBoost Classifier, Decision Tree Classifier, Gradient Boosting Classifier, K-Nearest Neighbors Classifier, LightGBM Classifier, Random Forest Classifier, and Extreme Gradient Boosting Classifier. Additionally, to illustrate the relationship between various factors and the delayed initiation of antenatal care, we employed the unsupervised machine learning approach known as the Apriori algorithm. After testing various classifiers on balanced training data, the LGBM Classifier emerged as the top performer for predicting delayed antenatal care initiation, achieving 81% accuracy and an area under the ROC curve of 81%.

### Model training

After selecting the model, we trained it using both balanced and unbalanced data and compared their performance through tenfold cross-validation. Following this comparison, the LightGBM model was chosen and subsequently trained with balanced data for the final prediction on unseen test data.

### Model evaluation

**Figure 3:**
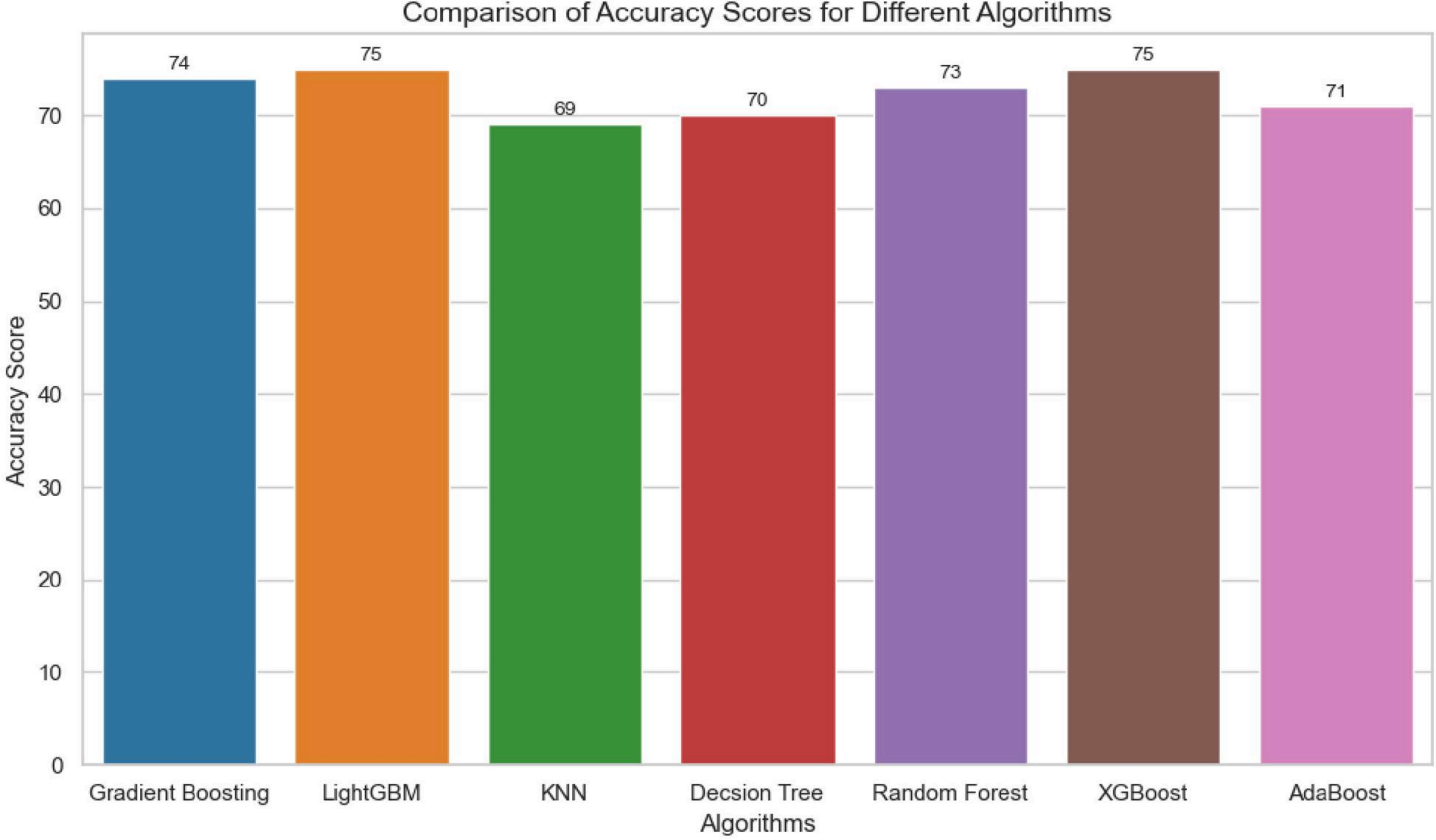
Model evaluation metrics after SMOTE.

**Figure 4:**
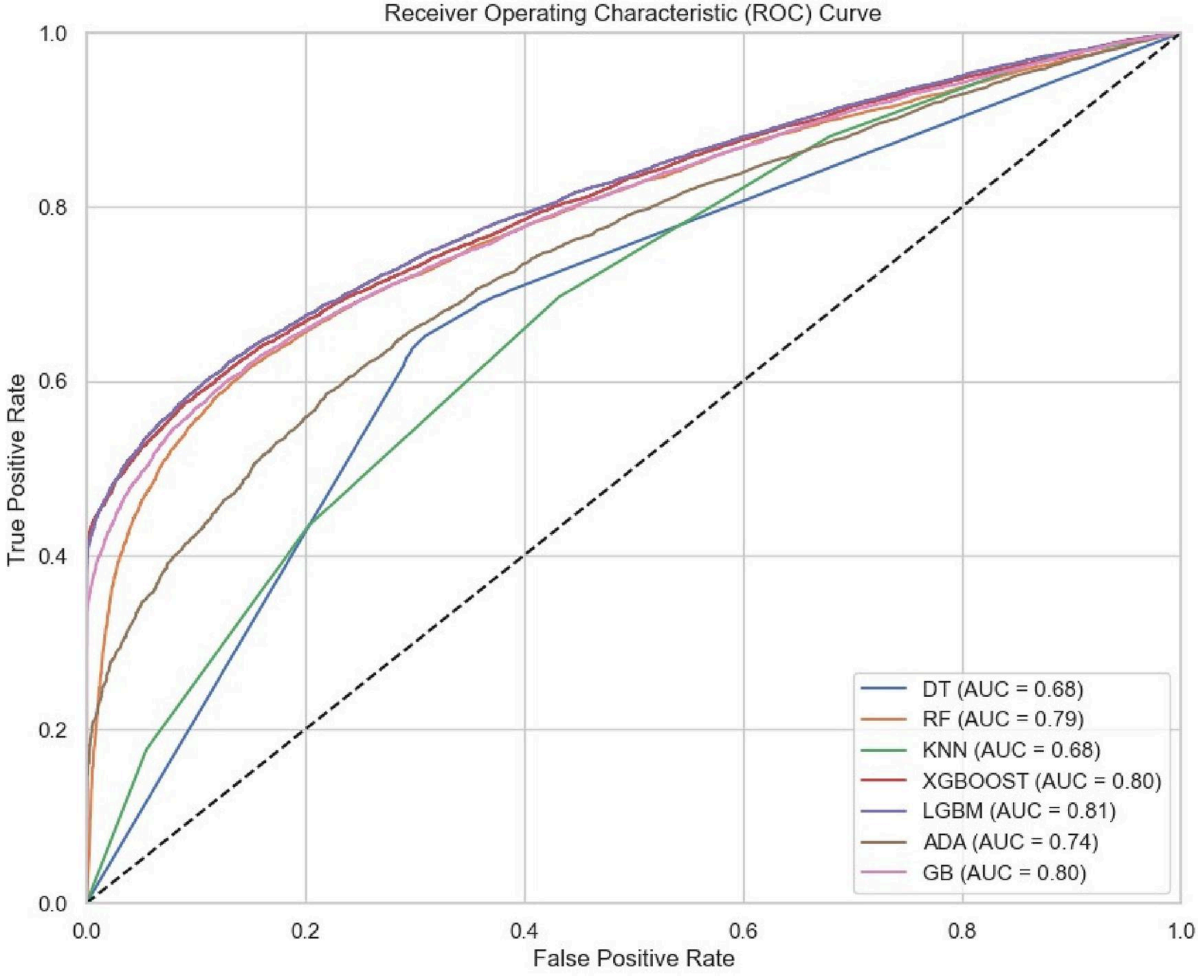
The model comparison on the test data.

Model evaluation is crucial to determine the model's strengths and weaknesses and test the model's capacity to generalize and yield precise predictions on data that hasn't been seen yet (55). We assessed the models' effectiveness in classifying delayed initiation of antenatal care using metrics such as accuracy, precision, recall, F1 score, and AUC- In this study, the LightGBM model emerged as the best performer, with an AUC-ROC of 81 and an accuracy of 75. Seen in Table 1 and Figures 3, 4.

**Table 1:** Model comparison through cross-validation on training.

Algorithm	Data	Accuracy (%)	AUC (%)	F1 Score (%)	Recall	Precision (%)
KNN classifier	Unbalanced	64	59	62	63	61
	Balanced	69	68	68	69	67
LGBM classifier	Unbalanced	65	60	63	64	62
	Balanced	75	81	78	79	77
DT classifier	Unbalanced	66	64	65	66	64
	Balanced	70	68	69	70	68
Random Forest	Unbalanced	73	57	65	64	66
	Balanced	73	79	75	76	74
XGB Classifier	Unbalanced	68	59	63	62	64
	Balanced	75	80	77	76	78
AdaBoost classifier	Unbalanced	68	58	63	62	64
	Balanced	68	51	60	59	61
Gradient Boost	Unbalanced	58	61	58	57	59
	Balanced	74	80	76	75	77

### Feature selection

Appropriate feature selection can enhance model performance by providing relevant data, reducing over fitting, speeding up training, and improving interpretability (56). We used Recursive Feature Elimination (RFE), a wrapper-based feature selection method, along with SHAP values to identify the most relevant features for our machine learning model Significant features are important for policymakers to have access to these important insights to inform their decision-making.

**Figure 5:**
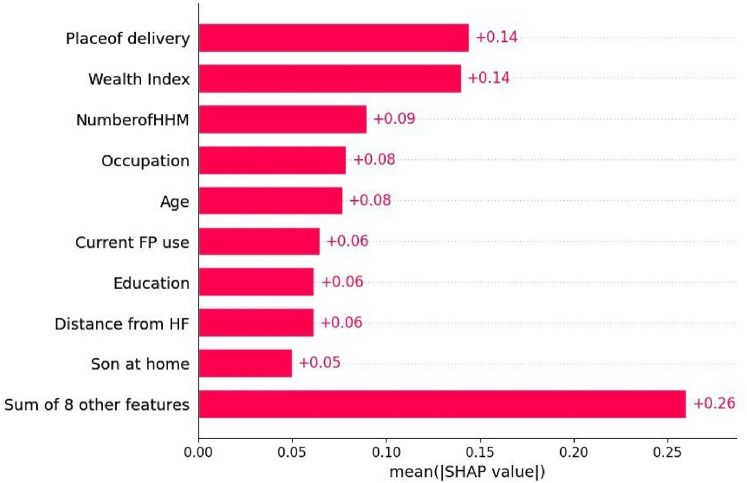
Feature selection methods for delayed antenatal care initiation.

We employed the Recursive Feature Elimination (RFE) method, and a wrapper-based feature selection approach, to identify the most pertinent features for our machine learning model. The performance of the ML model depends on the quality and the relevance of the features. Significant features are important for policymakers to have access to these important insights to inform their decision-making. As seen in Figure 5.

### Association rule mining

**Figure 6:**
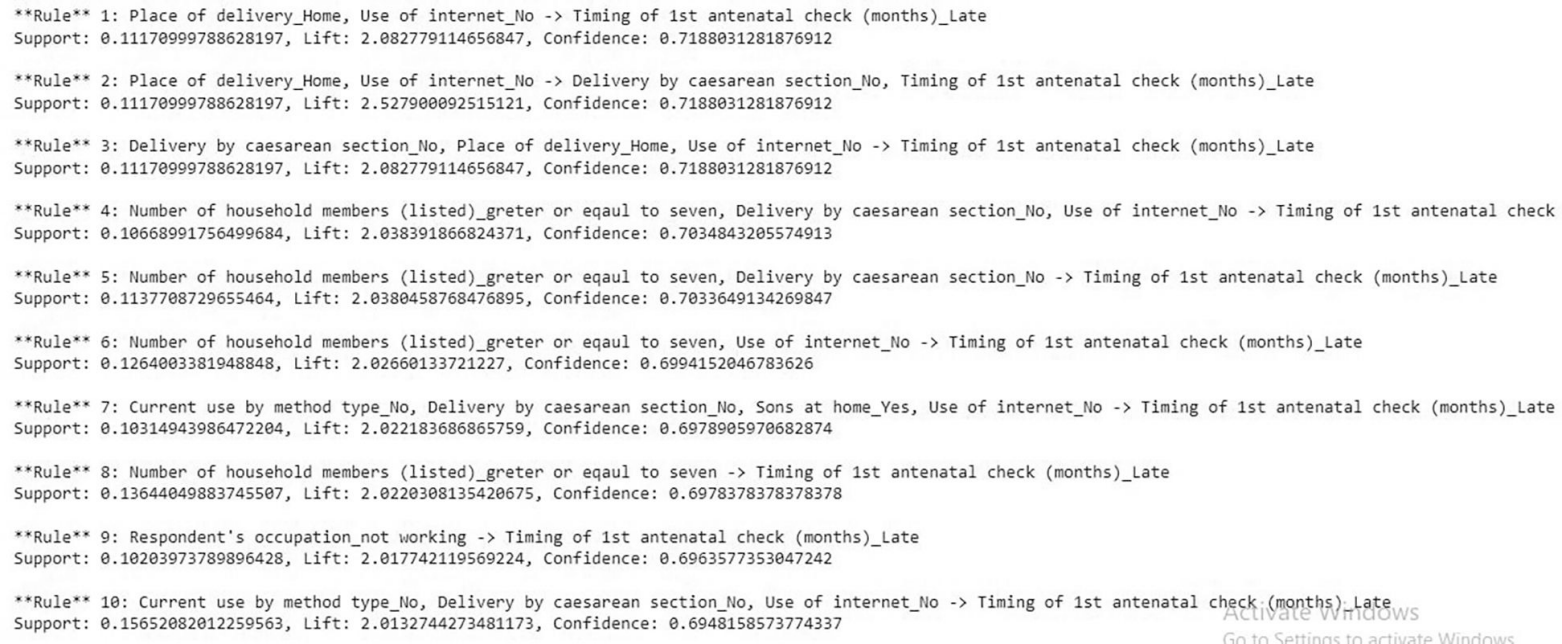
Association rule mining for predicting delayed antenatal care initiation among pregnant women.

Association rule mining is among the most important and popular data mining techniques, used for discovering hidden patterns and relationships based on specific confidence intervals and lift, thereby addressing limitations in feature selection (57) (Figure 6).

## Results

### Socio-demographic characteristics

This study contained a total of **77,865** (weighted) study participants. The majority of the study participants were from Burundi, 8879 (11.4%), Kenya, 9286 (11.9%), Malawi, 13,270 (17.0%), and Uganda, 9,957 (12.8%).

The majority of study participants, aged 20–24 and 25–29, accounted for approximately 25.3% (19,661) and 24.9% (19,412), respectively. The majority of the women lived in urban areas: 59,936 (77%). Additionally, 63,987 (82.2%) were married, while 24,872 (8.3%) were single. More than half of the study participants had primary education: 39,726 (51%). Seen in Table 2.

**Table 2:** Socio-demographic and economic characteristics of respondents.

Variable	Category	Weighted frequency	Timing of 1st antenatal check (months)	
			Late	Early
Country	Burundi	8,879 (11.4%)	4,650 (52.4%)	4,229 (47.6%)
	Zimbabwie	7,245 (9.3%)	4,554 (62.9%)	2,691 (37.1%)
	Ethiopia	4,771 (6.1%)	3,222 (67.5%)	1,550 (32.5%)
	Kenya	9,286 (11.9%)	6,422 (69.2%)	2,864 (30.8%)
	Madagascar	8,277 (10.6%)	5,393 (65.2%)	2,884 (34.8%)
	Malawi	13,270 (17.0%)	10,030 (75.6%)	3,240 (24.4%)
	Mozambique	4,726 (6.1%)	3,595 (76.1%)	1,131 (23.9%)
	Ruwanda	6,160 (7.9%)	2,463 (40%)	3,696 (60%)
	Tanzania	5,292 (6.8%)	3,269 (61.8%)	2,023 (38.2%)
	Uganda	9,957 (12.8%)	7,004 (70.3%)	2,953 (29.6%)
Age	15–19	6,027 (7.7%)	4,235 (70.3%)	1,792 (29.7%)
	20–24	19,661 (25.3%)	12,966 (65.9%)	6,696 (34.1%)
	25–29	19,412 (24.9%)	12,201 (62.8%)	7,212 (37.2%)
	30–34	15,389 (19.8%)	9,832 (63.9%)	5,557 (36.1%)
	35–39	10,731 (13.8%)	6,977 (65%)	3,754 (35%)
	40–44	5,107 (6.6%)	3,363 (65.9%)	1,744 (34.1%)
	45–49	1,537 (2%)	1,030 (67%)	507 (33%)
Place of residence	Urban	17,929 (23%)	11,113 (62%)	6,816 (38%)
	Rural	59,936 (77%)	39,490 (65.9%)	20,445 (34.1%)
Education	No education	15,042 (19.3%)	10,056 (66.9%)	4,986 (33.1%)
	Primary	39,726 (51%)	26,307 (66.2%)	13,419 (33.8%)
	Secondary	19,061 (24.5%)	12,247 (64.3%)	6,814 (35.7%)
	Higher	4,035 (5.2%)	1,993 (49.4%)	2,042 (50.6%)
Number of household members (list	1–6	54,080 (69.5%)	34,235 (63.3%)	19,845 (36.7%)
	≥7	23,785 (30.5%)	16,368 (68.8%)	7,416 (31.2%)
Sex of household head	Male	59,804 (76.8%)	38,691 (64.7%)	21,113 (35.3%)
	Female	18,060 (23.2%)	11,912 (66%)	6,148 (44%)
Currently marital status	Single	5,640 (7.2%)	3,836 (68.0%0	1,803 (32%)
	Married	63,987 (82.2%)	41,305 (64.6%)	22,682 (35.4%)
	Widowed	1,177 (1.5%)	780 (66.3%)	397 (33.7%)
	Divorced	7,061 (9.1%)	4,682 (66.3%)	2,379 (33.7%)
Sons at home	No	20,819 (26.7%)	13,065 (62.8%)	7,754 (37.2%)
	Yes	57,046 (73.3%)	37,538 (65.8%)	19,507 (34.2%)
Distance to a health facility	Big problem	28,427 (36.5%)	19,600 (68.9%)	8,837 (31.1%)
	Not a big problem	49,427 (63.5%)	31,003 (62.7%)	18,424 (37.3%)

### Socioeconomic-related characteristics

The study included a total of 77,865 individuals (weighted). Of these, 68,035 (87.4%) had no internet access, and 34,036 (43.6%) had a mobile phone. Among those who were able to read, 51,967 (66.5%) did so, while approximately 18,039 (23.1%) had the poorest wealth index, and 54,526 (69.8%) of women were working. Seen in Table 3.

**Table 3:** Socio-economic characteristics of respondents.

Variable	Category	Weighted frequency	Timing of 1st antenatal visits (months)		
			Late	Early	Total
Use of internet	No	68,035 (87.4%)	45,081 (66.3%)	22,953 (33.7%)	68,034
	Yes	9,830 (12.6%)	5,522 (56.2%)	4,308 (43.8%)	9,830
Mobile phone	No	44,825 (57.6%)	30,184 (67.3%)	14,641 (32.7%)	44,825
	Yes	33,040 (42.4%)	20,420 (61.8%)	12,620 (38.2%)	33,040
Literacy	Cannot read at all	25,492 (32.7%)	17,324 (68%)	8,168 (32%)	25,492
	Able to read	52,372 (67.3%)	33,279 (63.5%)	19,093 (36.5%)	52,372
Wealth index status	Poorest	16,696 (21.4%)	11,342 (67.9%)	5,354 (32.1%)	16,696
	Poorer	15,792 (20.3%)	10,641 (67.4%)	5,151 (32.6%)	15,792
	Middle	15,109 (19.4%)	10,086 (66.8%)	5,024 (33.2%)	15,110
	Richer	15,393 (19.8%)	10,070 (65.4%)	5,324 (34.6%)	15,394
	Richest	14,874 (19.1%)	8,465 (56.9%)	6,409 (43.1%)	14,874
Occupation	Not working	22,822 (29.3%)	15,681 (68.7%)	7,141 (31.3%)	22,822
	Working	55,043 (70.7%)	34,923 (63.4%)	20,120 (36.6%)	55,043

### Maternal health service utilization and fertility-related factors

The study involved 77,865 individuals (weighted), with 49.0% using current methods, and 78.8% delivering at health facilities. Most participants (91.8%) were delivered by C-section, and about 60.4% had 1 to 3 children. see in Table 4.

**Table 4:** Maternal health service characteristics of respondents.

Variable	Category	Weighted frequency	Timing of 1st antenatal check (months)		
			Late	Early	Total
Current use by method type	No	39,749 (51.0%)	26,679 (67.1%)	13,070 (32.9%)	39,749
	Yes	38,115 (49.0%)	23,925 (62.8%)	14,191 (37.2%)	38,116
Place of delivery	Home	16,281 (20.9%)	11,998 (73.7%)	4,283 (26.3%)	16,281
	H/Facility	61,583 (79.1%)	38,605 (62.7%)	22,978 (37.3%)	61,583
Delivery by CS	No	71,475 (91.8%)	47,211 (66.1%)	24,264 (33.9%)	71,475
	Yes	6,390 (8.2%)	3,393 (53.1%)	2,997 (46.9%)	6,390
Total children ever born	1–3	47,638 (61.2%)	29,878 (62.7%)	17,760 (37.3%)	47,638
	4–6	21,660 (27.8%)	14,612 (67.5%)	7,048 (32.5%)	21,660
	7–9	7,130 (9.2%)	5,075 (71.2%)	2,055 (28.8%)	7,130
	>9	1,437 (1.8%)	1,039 (72.3%)	398 (27.7%)	1,437

### Overall initiation of the first antenatal care visit

**Figure 7:**
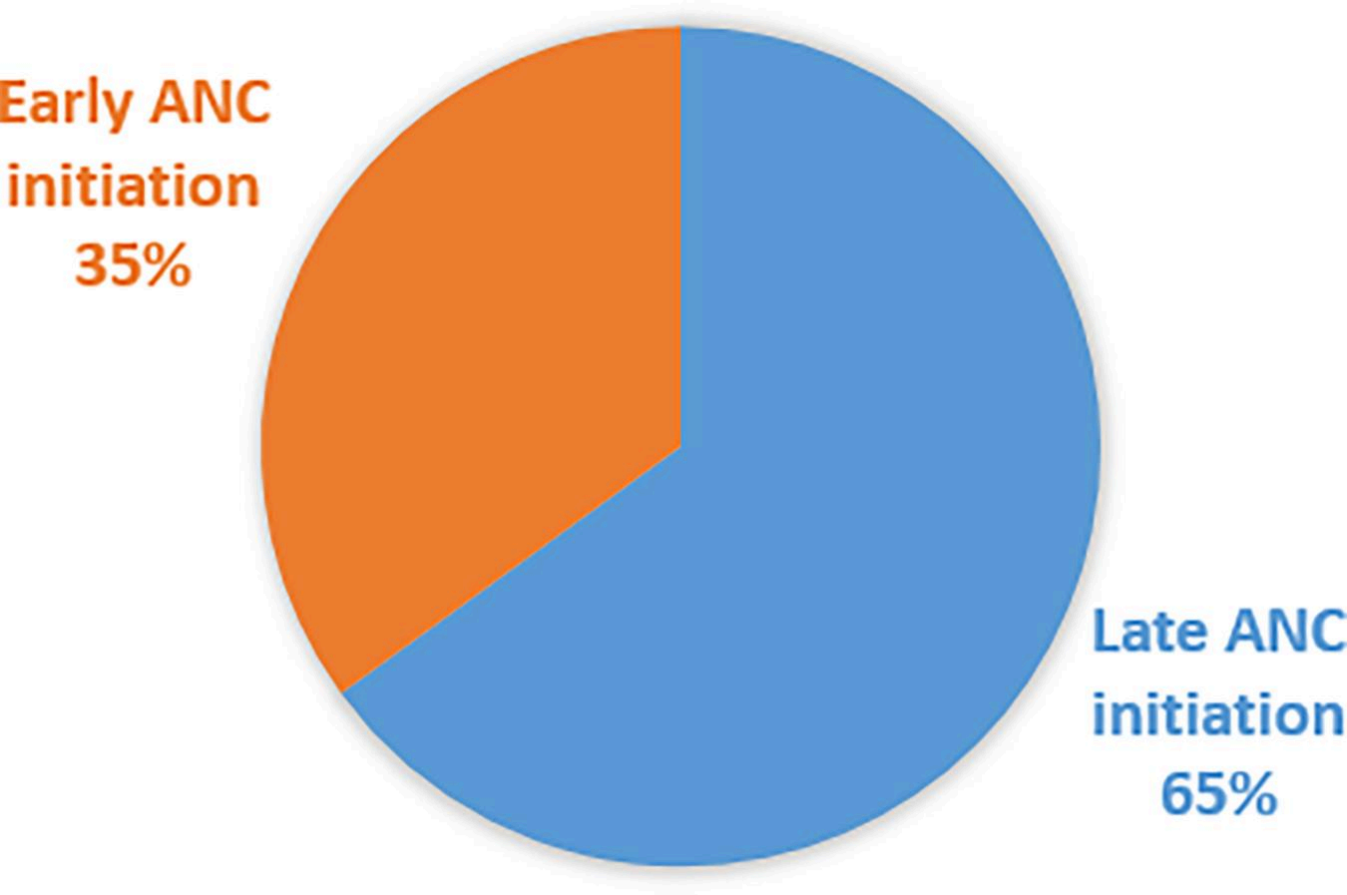
Overall initiation of antenatal care visits.

Out of the total, only 35% of pregnant women initiated ANC early (Figure 7).

### Association rule mining

Apriori algorithms:

Rule 1: If place of delivery = home, internet access = no, Then the probability of delay antenatal care initiation is 71.8% (lift = 2.8).
Rule 2: If place of delivery = home, internet access = no, delivery by Cesarean section = no, Then the probability of delayed antenatal care initiation is 71.8% (lift = 2.5).
Rule 3: If number of HH member >7 delivery by Cesarean section = no, internet = no, Then the probability of delayed antenatal care initiation is 70% (lift = 2.03).
Rule 4: If number of HH member >7 internet access = no, Then the probability of delayed antenatal care initiation is 69% (lift = 2.02).
Rule 5: If number of HH member >7 delivery by Cesarean section = no, Then the probability of delayed antenatal care initiation is 70% (lift = 2.03).
Rule 6: If current use by method = no, delivery by Cesarean section = no, sons at home = yes, internet access = no, Then the probability of delayed antenatal care initiation is 69% (lift = 2.02).
Rule 7: If number of HH member > 7 Then the probability of delayed antenatal care initiation is 69% (lift = 2.02).
Rule 8: If respondent occupation = not work Then the probability of delayed antenatal care initiation is 69% (lift = 2.01).
Rule 9: If current use by method = no, delivery by Cesarean section = no, internet access = no, Then the probability of delayed antenatal care initiation is 69% (lift = 2.01).

## Discussion

This study aims to identify the top predictors of delayed antenatal care initiation in East Africa, including Ethiopia, to provide input for policymakers. The LGBM model demonstrated superior performance compared to other classifiers, achieving an accuracy of 75%, an AUC score of 81%, an F1 score of 78%, a recall of 79%, and a precision of 77%. The performance of the LGBM model is supported by previous machine learning studies (58, 59). Its superior performance can be attributed to its efficiency, advanced learning strategy, ability to capture complex patterns, and better generalization. LGBM identified.

In this study, the overall delayed initiation of antenatal care in ten East African countries is 65% which is in line study conducted in Afghanistan (66.9%) (29), and Mozambique (60%) (31). However, higher than the studies conducted in some parts of Ethiopia (47%, 58%, 59%) (32–34), New Zealand (17%) (27), and Gambia (56%) (28), lower than a study done in Malawi (75.6%) (30).

A possible explanation for this difference could be that this study encompasses a broader scope, covering ten countries, whereas previous studies focused on a single country.

This study found that women who delivered at home had an increased likelihood of initiating antenatal care lately. This is supported by previous studies (38, 60, 61). The possible explanation for this finding could be that women who deliver at home often have lower awareness about the importance and timing of antenatal care (ANC), and may not fully understand the potential complications and risks to themselves and their babies associated with missing ANC. Additionally, the majority of women who deliver at home live in rural areas and have a low level of education.

Also, the study showed that women from poor households had an increased likelihood of delayed initiation of antenatal care. This is supported by previous studies (62, 63). The possible explanation is that women from poor families tend to give less attention to healthcare-seeking behaviors, as they are often more focused on meeting basic living needs. As a result, the probability of early antenatal care initiation is low among this group.

This study found that having a large number of household members, increases the likelihood of late initiation of antenatal care (ANC), this is supported by previous studies (47, 61, 64). The possible explanations could be women from larger households may experience increased domestic responsibilities, such as caring for children, elders, or managing household tasks, which can limit their time and ability to seek health services early in pregnancy.

This finding also showed that women with no occupation had an increased likelihood of late initiation of antenatal care (ANC), and this is supported by previous studies (31, 42, 65) The possible explanation could be women with no occupation may delay ANC due to financial dependence, limited decision-making power, and lower exposure to health information, which reduces awareness of the importance of early care.

This finding showed that being a younger woman increases the likelihood of late initiation of antenatal care (ANC), this is supported by previous studies (32, 66, 67). Younger women, especially adolescents, may delay ANC due to lack of awareness about the importance and timing of antenatal care. They are also more likely to face social stigma, especially in cases of unintended or early pregnancies, making them reluctant to seek care early. Additionally, they often have limited autonomy in health decision-making and may depend on others financially or socially, which can further delay ANC initiation.

Women who do not use family planning methods are woman increases the likelihood of late initiation of antenatal care, this is supported by previous evidence (66, 68, 69). The possible explanation for this could be unplanned pregnancies, limited awareness of maternal health, and reduced engagement with healthcare services.

Women with a low level of education are increases the likelihood of late initiation of antenatal care, as supported by previous studies (34, 38, 61, 70). Women with lower education levels often have limited knowledge about the importance and timing of antenatal care. They may not be fully aware of the benefits of early ANC in detecting and preventing pregnancy-related complications. Additionally, lower educational attainment is frequently associated with reduced decision-making power, low health literacy, and less access to health information.

This study also shows that women who live far from health facilities have an increased likelihood of late initiation of antenatal care, this is supported by previous evidence (16, 37, 38). The explanation for this finding could be that traveling long distances can take hours or a full day, making it difficult for women who have household duties. Financial constraints, including both direct and indirect costs of accessing health care services, increase with distance from health facilities. In addition, women living in rural areas may have lower levels of awareness and limited practice of visiting health facilities.

This finding shows that having a son at home increases the likelihood of delayed initiation of antenatal care (71). Having a son at home might reduce a woman's perceived need for early antenatal care, either due to cultural satisfaction, prior positive pregnancy experience, or competing household responsibilities.

### Strength and limitation of the study

Our findings interpreted with consideration of certain limitations associated with the use of secondary data, as it may introduce biases that could impact the overall generalizability of the results. The DHS data lacks several key behavioral variables that could influence ANC initiation, and it is not designed to support causal inference. Under the strengths section, using hybrid algorithms that combine both supervised and unsupervised learning approaches enhances the accuracy, robustness, and adaptability of models, making them valuable tools in this study and cover wide stud area.

## Conclusion

The prevalence of late antenatal care (ANC) initiation is high, at 65%, in the study area. Home delivery, low household income, a large number of household members, unemployment, being a younger woman, not using family planning methods, low educational level, and rural residence are predictors of delayed antenatal care initiation.

Promote family planning utilization, targeted health literacy campaigns, youth-friendly programs, expand mobile clinics, and promote skilled birth attendance increase timely ANC initiation are recommended.

## Data Availability

The original contributions presented in the study are included in the article/Supplementary Material, further inquiries can be directed to the corresponding author.
